# Buffalo Medical College, Commencement

**Published:** 1864-03

**Authors:** 


### EDITORIAL DEPARTMENT.

### BUFFALO MEDICAL COLLEGE, COMMENCEMENT.

The Commencement exercises in the Buffalo Medical College were held on the evening of February 23d, when the degree of Doctor in Medicine was conferred upon the following gentlemen:

Edwin Bassett Thefft, Fort Miller, Washington county, New York.
Daniel Augustus Currie, Searsville, Orange county, New York.
John Mason Brown, Aurora, Erie county, New York.
Cyrus Emery Stebbins, Butternut, Otsego county, New York.
Thomas Benton Eagle, Loudonville, Ashland county, New York.
Samuel Payne Ford, Peterboro, Peterboro county, Canada West.
Newell Allen Dryer, Lansing, Ingham county, Michigan.
Oscar Henry Adams, Vanetten, Chemung county, New York.
Robert Taylor, Loundonville, Ashland county, New York.
Evan Gabriel Williams, Remsen, Onieda county, New York.
George McKnight, Sterling, Cayuga county, New York.
Oakman Sprague Payne, Rochester, Monroe county, New York.
Gershom Wells Hamilton, Mesopotamia, Trumbull county, Ohio.
Willis DeLancy Hall, Lockport, Niagara coounty, New York.
C· H. Otto Burger, Buffalo, Erie county, New York.

Alexis Edwin Williard, Friendship, Alleghany county, New York.
Daniel David Halsted, St. Charles, Saginaw county, Michigan.
Robert Renfrew Smith, Komoka, Middlesex county, Canada West.
Henry Martyn Gale, Ashville, Chatauqua county, New York.
Richard Reuben Carr Bordwell, Pen Yan, Yates county, New York.
Judson Aaron Gillet, Albion, Orleans county, New York.
Thomas Morford Brown, Clarkesville, Mercer county, Pennsylvania.
Lafayette Balcom, Ransomville, Niagara county, New York.
Ira L. Jones, Pulaski, Oswego county, New York.
Merrill Eugene Shaw, Buffalo, Erie county, New York.
Byron Thorne Wheeler, Canandaigua, Ontario county, New York.
Newton Loren Clark, Royalton, Niagara county, New York.
James Stratton Harlow, Bath, Stuben county, New York.
George Duryee, Buffalo, Erie county, New York.
John Moran Flood, Elmira, Chemung county, New York.
Francis Lucas Nesbitt, Oxford county, Canada West.
Hiram Dana Walker, ———, Cattaraugus county, New York.
Clayton Lewis Hill, Lafayette, Onondaga county, New York.
James Nichols, Limestone, Cattaraugus county, New York.
George Thompson, Humberstone, Welland County, New York.
Aralin Hubert Street, Barre Centre, Orleans county, New York.
Hiram Dexter Kee, Greensburgh, Trumbull county, Ohio.
Richard Jones, Prince Albert, Ontario county, Canada West.
Johnathan Edwards, Macomb, Macomb county, Michigan.
William Henry Gail, Willink, Erie county, New York.
Henry Van Alstyne Mott, Oreville, Butts county, California.

The Honorary degree of M. D. was conferred upon Eber Smith Carlisle, of Plessis, Jefferson, Co. N. Y.

The following Physicians were appointed Curators for four years:

D. P. Bissell, Utica, Oneida county, N, Y.; T. Nesbitt, M. D., Avon, Livingston county, N. Y.; Dr. Landercale, Geneseo, Livingston county, N. Y.

The Faculty deem the following Theses worthy of honorable mention, viz:

A Thesis on "Dynamics of Inflammation," by George McKnight, of Sterling, Cayuga county, New York.

A Thesis upon "The Philosophy of Cure," by Samuel P. Ford, of Peterboro, Canada West.

A Thesis on "Thoracic Aneurism," by Edwin B. Thefft, A. B., of Fort Miller, Washington, county, New York.

The Valedictory was delivered by Edwin B. Thefft, a member of the

Graduating Class. Prof. Charles A. Lee gave the charge to the Graduates. The exercises were interesting and instructive, and were attended by a large and respectable audience.

Thus closed one of the most prosperous terms ever enjoyed by the University of Buffalo, graduating some forty intelligent, well educated physicians, who, we believe, are to fill places of honor and responsibility, doing credit to themselves and reflecting lustre upon the Institution from which they have graduated.

After the commencement exercises, the Faculty invited the Curators to supper at the American Hotel, where, after full satisfaction of the inner man, the higher intellectual nature also received attention. Prof. Eastman, Dean of the Faculty, spoke of the aims and objects of the College, saying that it was the design to give thorough and complete instruction in every department of medical knowledge, and to elevate the standard of medical education. He invited the Curators to assist them in the effort to advance the standard of acquirement, and thanked them, in behalf of the Faculty, for their attendance and hearty co-operation; hoping to meet them upon this annual annniversary of the College, and ever to merit their hearty approval. Spoke of the importance of physicians not admitting young men to their offices as students of medicine who had not thorough preliminary preparation.

Dr. Miner was called upon in connection with the *Medical Journal.* He said he had nothing especial to communicate on the occasion, so far as the *Journal* was concerned: it spoke monthly for itself, and must stand upon its own merits. He had deep interest in it, and was always glad to talk about it upon all proper occasions; never ceasing to remind the profession, that periodical medical literature was an "institution" of itself, and should receive its earnest consideration. This was not the topic upon which he proposed to remark, but had risen to reply to the Dean, who had said, that it was the design of the Faculty to make every effort to advance the standard of medical education. This he believed to be true, and would congratulate the Faculty of the University of Buffalo upon having re-proposed and urged the most important measure of reform, calculated, if adopted, to revolutionize the present plan of granting diplomas, and give the profession a security and a protection which it has never enjoyed in this country. The appointment of a State Board of Examiners, independent of teaching, and impartial, was a measure which should receive the approval of all interested in the honor and usefulness of the profession. It was an intimation on the part of the Buffalo College, so far as it might have been selfishly considered, that she was willing to place her students upon an impartial trial, side by side with those of her sister institutions. It was evidence that it was not her purpose or pride to make yearly a great number of doctors, but to make educated, capable physicians who, in doing themselves honor, should honor her. He did not wish to so heartily approve this measure as to make it appear that he regarded Teachers and Curators as altogether unsuitable persons to make examinations and grant diplomas, but he did desire to congratulate the Faculty of the Buffalo College upon being foremost in re-proposing and advocating, in tangible form, the most important measure for the advancement of the highest interests of the profession—of doing this even at their own sacrifice; for it was manifestly liable to diminish rather than augment the number of medical students. This constituted, in some degree, the grandeur of the action, proving the purity of the motive. He believed that the profession would everywhere rise up to thank them, for the part they have acted in the initiation of this measure.

Prof. Lee remarked, that as he had been instrumental in proposing this measure, and had presented it to the State Medical Society, it would not be inappropriate for him to reply to Dr. Miner's sentiments of approval and congratulation. He had found that distinguished teachers in the colleges of New York regarded the diploma, as awarded by themselves and others, as simply a certificate that the possessor had complied with the requirements of the law; that is, that they had studied three years and attended two courses of medical lectures; that nothing more than this could be said of it. The custom had become universal in many institutions of graduating all applicants after the terms of the law had been complied with. So unexceptionable was this, that classes of a hundred or more had been approved without a single rejection. He had expected opposition to the measure, but, to his surprise, none had yet been offered. It would be presented to the American Medical Association, at its meeting in New York City, in June next, when probably it would meet with opposition, but not sufficient to prevent its adoption. The State Board should be paid without levy upon the student; should be independent of all colleges, and make uniform requirements, thus constituting an impartial tribunal, before which all might be admitted, and if found worthy, receive approbation and a diploma, which would be an indication of merit, and would pass everywhere as evidence of qualification, and not merely as a certificate of having spent three years in the office of a physician and of having attended two courses of lectures. He regarded this movement as sure eventually to succeed and work a revolution in this country which would be of immense benefit in raising the standard of qualification and of greatly increasing the value of the degree of Doctor in Medicine. In Europe this plan had long been adopted, and an impartial and uniform Board was empowered to grant diplomas. He hoped the same plan would soon be inaugurated here, when a more actual value would properly attach to a medical diploma.

Prof. White made mention of the distinguished absent ex-members of the Faculty of the University of Buffalo, and of the prominent places now occupied by them, as teachers, authors, or otherwise. Three distinguished teachers of Physiology in this country had been connected with the Buffalo College, and two of them, Dalton and Flint, Jr., had gone out from it while Mason still remained to teach Physiology by vivisections, these three being the only teachers in the country who illustrate and enforce their teachings by this method. Others not less distinguished had been connected with the Faculty, but are now engaged in other fields. Would propose as a sentiment: The success, happiness, and increasing fame of the ex-members of the Faculty of the University of Buffalo.

Prof. Lee said it should be remembered, to the credit of this College, that three of the best and most popular Text Books in our language, or in any langnage, had been written by members of this Faculty, and the material obtained mostly while holding this connection and in Buffalo: Hamilton on Fractures and Dislocations; Flint on Diseases of the Chest; Dalton's Physiology, were three books which were unsurpassed in merit by any works in any country upon these subjects.

Prof. Eastman spoke to the memory of a deceased Curator, Dr. Charles H. Wilcox, and gave incidents in his life, and prominent traits in his character, holding his memory in most pleasant remembrance. This sentiment was drank standing, and in silence.

Prof. Rochester spoke also of his life and character, and made pleasant reference to his good qualities of head and heart, devotion to his profession, high sense of professional honor, rectitude of life and benevolence of purpose.

Sentiments were given by Drs. Lothrop, Lockwood, Strong, of Westfield, Baker, of Warsaw, Root, of Batavia, and others, and the best spirit of harmony and professional courtesy prevailed. There was nothing to interrupt the current of high social entertainment, and we have do doubt that the Faculty of the College, as well as the Curators, many of whom had taken much pains to be present, were highly gratified with the social opportunity.

