# Correction

**Published:** 1864-03

**Authors:** 


Correction.—Our compositor insists upon spelling "Tefft," in our report of Commencement Exercises, with an "h," giving the name an accent of dishonesty. We assure our readers that it only gives the name an "Irish brogue," and was wholly unintentional. "Repentance comes, alas! too late for reform." Other names in the list of graduates may have suffered, and equally require correction.

